# The Effect of Cordycepin on Steroidogenesis and Apoptosis in MA-10 Mouse Leydig Tumor Cells

**DOI:** 10.1155/2011/750468

**Published:** 2011-06-05

**Authors:** Bo-Syong Pan, Chun-Yu Lin, Bu-Miin Huang

**Affiliations:** Department of Cell Biology and Anatomy, College of Medicine, National Cheng Kung University, Tainan 701, Taiwan

## Abstract

Cordycepin is a natural pure compound extracted from *Cordyceps sinensis* (CS). We have demonstrated that CS stimulates steroidogenesis in primary mouse Leydig cell and activates apoptosis in MA-10 mouse Leydig tumor cells. It is highly possible that cordycepin is the main component in CS modulating Leydig cell functions. Thus, our aim was to investigate the steroidogenic and apoptotic effects with potential mechanism of cordycepin on MA-10 mouse Leydig tumor cells. Results showed that cordycepin significantly stimulated progesterone production in dose- and time-dependent manners. Adenosine receptor (AR) subtype agonists were further used to treat MA-10 cells, showing that A_1_, A_ 2A _, A_ 2B _, and A_3_, AR agonists could stimulate progesterone production. However, StAR promoter activity and protein expression remained of no difference among all cordycepin treatments, suggesting that cordycepin might activate AR, but not stimulated StAR protein to regulate MA-10 cell steroidogenesis. Meanwhile, cordycepin could also induce apoptotic cell death in MA-10 cells. Moreover, four AR subtype agonists induced cell death in a dose-dependent manner, and four AR subtype antagonists could all rescue cell death under cordycepin treatment in MA-10 cells. In conclusion, cordycepin could activate adenosine subtype receptors and simultaneously induce steroidogenesis and apoptosis in MA-10 mouse Leydig tumor cells.

## 1. Introduction

*Cordyceps sinensis* (CS) is an ingredient of traditional Chinese medicine and is prescribed for replenish the kidney and soothe the lung and for the treatment of fatigue [1]. Cordycepin (3′-deoxyadenosine, an adenosine analogue) is a pure component extracted from the mycelia of CS, and it is well known to possess anticancer ability which induce apoptosis in HeLa cells, oral cancer cells, breast cancer cells, leukemia, and lymphoma cell lines [2–5]. Previous studies demonstrated that CS alone could stimulate steroid production in both normal and tumor mouse Leydig cells [6, 7] and activate apoptosis in MA-10 mouse Leydig tumor cells [8]. It is highly possible that cordycepin is the main component in CS modulating Leydig cell functions. Thus, the aim of the present study was to investigate the steroidogenic and apoptotic effects with potential mechanism of cordycepin on MA-10 mouse Leydig tumor cells.

Steroidogenesis, steroid hormone biosynthesis, occurs mainly in the adrenal glands, brain, placenta, testes, and ovaries [9]. In the male reproduction system, steroidogenesis in Leydig cells is regulated by luteinizing hormone (LH)/human chorionic gonadotropin (hCG). LH and hCG activate its cognate receptors and coupling to the adenylate cyclase (AC) through the heterotrimeric guanine nucleotide-binding regulatory protein (G-protein) [10, 11]. The activated GTP-bound *α* subunit of G-protein would be able to activate adenylyl cyclase, which results in the hydrolysis of ATP to cyclic AMP. Once cAMP is synthesized, the following activation of protein kinase A (PKA) pathway would phosphorylate steroidogenic acute regulatory protein (StAR) [12]. The StAR protein, a 30 kDa phosphoprotein, is the rate-limiting step which delivers cholesterol from the outer to the inner mitochondrial membrane [13]. After translocation into mitochondrial, P450 side chain cleave enzyme (P450scc) converts cholesterol to pregnenolone [14]. When pregnenolone is formed, it may be metabolized to progesterone by mitochondrial 3*β*-hydroxysteriod dehydrogenase (3*β*-HSD), or it may exit the mitochondria and undergo further metabolism with the final steroid hormone product dependent upon the nature of the tissue [15, 16].

It has been well demonstrated that adenosine acts through four G-protein-coupled membrane receptors, the A_1_, A_2A_, A_2B_, and A_3_ adenosine receptors [17]. The adenosine A_1_ and A_3_ receptors (A_1_-AR and A_3_-AR) inhibit adenylate cyclase via G_i_ and activate phospholipase C (PLC) [18, 19], and the adenosine A_2A_ and A_2B_ receptors (A_2A_-AR and A_2B_-AR) stimulate adenylyl cyclase via G_s_ [20, 21], respectively. It is also shown that adenosine appears to induce cell death through apoptosis, whereas ATP appears to cause both necrosis and apoptosis [22]. 

Although some reports have showed that cordycepin possesses anticancer ability, there is still no research about cordycepin on gonadal steroidogenesis. In fact, we have demonstrated that cordycepin could stimulate testosterone production in normal mouse primary Leydig cells without any phenomenon of cell death [23]. Since the isolation of primary Leydig cell is complicated with very low yield from mouse testis, MA-10 mouse Leydig tumor cell line was then used to further examine the regulatory mechanisms regarding the effects of cordycepin. Interestingly, we did find that cordycepin could induce MA-10 cell steroidogenesis and apoptosis. Thus, our objective was to investigate the effect of cordycepin on Leydig cell steroidogenesis and apoptosis with the preliminarily possible mechanisms.

## 2. Materials and Methods

### 2.1. Chemicals

Cordycepin, bovine serum albumin (BSA), Waymouth MB 752/1 medium, *N*6-cyclopentyladenosine (CPA), 2-*p*-(2-carboxyethyl) phenethylamino-5′-*N*-ethyl-carboxamidoadenosine (CGS-21680), 5′-*N*-ethylcarboxamidoadenosine (NECA), N6-(3-iodobenzyl) adenosine-5′-N-methyluronamide (IB-MECA), 8-Cyclopentyl-1,3-dipropylxanthine (DPCPX), 8-(3-Chlorostyryl) caffeine (CSC), 8-[4-[((4-Cyanophenyl)carbamoylmethyl)oxy]phenyl]-1,3-di(n-propyl) xanthine (MRS 1754), 3-Ethyl-5-benzyl-2-methyl-4-phenylethylethynyl-6-phenyl-1,4-dihydropyridine-3,5-dicarboxylate (MRS 1191), human chorionic gonadotropin (hCG) (10,000 IU per gram) and methylthiazolecterazolium (MTT) were purchased from Sigma Chemical (St. Louis, mo, USA). Fetal bovine serum (FBS), Dulbecco's phosphate-buffered saline, lyophilized trypsin-EDTA, and gentamicin sulfate were purchased from Gibco (Grand Island, NY, USA). Sodium hydroxide, hydrochloric acid, Sodium dodecyl sulfate (SDS), sucrose, EDTA, isopropyl alcohol, chloroform, and Tween 20 were purchased from Merck (Darmstadt, Germany). Tris base was purchased from Calbiochem (San Deigo, Calif, USA). Acrylamide was purchased from J.T. Baker (Phillipsburg, NJ, USA). HEPES was purchased from Mallinckrodt Baker, Inc. (Paris, USA). Tissue culture grade sodium bicarbonate, sodium carbonate, sodium chloride, sodium dihydrogen phosphate, and potassium chloride were purchased from Riedel-deHaen (Seelze, Germany). dNTP and MMLV reverse transcriptase were purchased from Promega (Madison, Wis, USA). Taq was purchased from ABgene (Surrey, UK). Donkey antirabbit IgG conjugated with horseradish peroxidase were purchased from Amersham International (Arlington Heights, Ill, USA). Antibody against *β*-actin was purchased from Cell Signaling (Beverly, USA). Antibody against StAR was a generous gift from Dr. Strauss (University of Pennsylvania Medical Center, USA). Charcoal was purchased from Showa Chemical Inc. (Tokyo, Japan). [^3^H] progesterone used for radioimmunoassay was purchased from Dupont-New England Nuclear (Boston, USA). Antiserum to progesterone was a kind gift from Dr. Paulus S. Wang (National Yang Ming University, Taipei, Taiwan).

### 2.2. Cell Culture

The MA-10 cell line was a gift from Dr. Mario Ascoli (The University of Iowa, Iowa City, USA) and is maintained at 37°C in a humidified environment containing 95% air and 5% CO_2_ for all the following experiments. MA-10 cells (5 × 10^3^ cells/100 *μ*L medium) or (6 × 10^5^ cells/2 mL medium) were plated into 96-well plates or 6 cm dish grown for 24 hr in Waymouth medium containing 10% fetal bovine serum, respectively. The medium was removed and the cells were washed twice with 1X PBS, and then treated with various concentrations of cordycepin in serum free Waymouth medium for indicated time periods. The cells were then isolated for total protein. The expression of target protein was determined by Western blot analysis. Cytotoxicity assay and cell morphological analysis of the MA-10 cells were determined by MTT assay. The media were withdrawn and progesterone levels were determined by radioimmunoassay. Finally, the adenosine receptor subtypes mRNA expression were performed by RT-PCR.

### 2.3. Morphology Study

MA-10 cells were seeded in 96-well plate (Techno Plastic Products AG, Trasadingen, Switzerland) containing 5 × 10^3^ cells with 100 *μ*L serum medium in each well. After 70–80% confluence, cells were treated without or with 100 *μ*M and 1 mM cordycepin for 24 hr, respectively. Cell morphology was then observed and recorded under light microscopy (Olympus, CK40). Apoptosis was characterized by the loss of cellular contact with the matrix and the appearance of plasma membrane blebbing.

### 2.4. MTT Cytotoxicity Assay

Cordycepin-induced MA-10 cells cytotoxicity was determined by measuring mitochondrial succinate dehydrogenase activity using a modification of the MTT assay. After MA-10 cells reach 70–80% confluence, cells were treated with serum free medium containing 100 *μ*M and 1 mM cordycepin for indicated time points (24 and 48 hr). MTT was added to wells (0.5 mg/mL final concentration) after time points of culture at 37°C in a 5% CO_2_ humidified atmosphere in the presence of the desired reagents. Mitochondrial dehydrogenases of viable cells will convert MTT into a color-dense formazan. Four hours later, the DMSO was added in wells to dissolve the formazan. The DMSO solutions were added and the absorbance was measured at 590 nm by an ELISA reader (VersaMax, MDS Inc., Toronto, Canada).

### 2.5. DNA Fragmentation Assay

Cells (1 × 10^6^) were lysed in a 0.6 mL cell lysis solution containing 20 mM Tris-HCl, 10 mM EDTA, pH 8.0 and 0.3% Triton X-100. DNA was extracted with 0.6 mL phenol/chloroform (1 : 1), and the mixture was centrifuged at 12,500 rpm for 10 min. DNA in the aqueous phase was extracted with phenol/chloroform (1 : 1) again. The aqueous (DNA containing) phase was mixed with isopropanol at −20°C overnight. After centrifugation, DNA pellets were washed with 70% ethanol and air-dried. DNA pellets were dissolved in TE buffer (10 mM Tris-HCl, 1 mM EDTA, pH 8.0), and RNase A (3 mg/mL) was added to remove RNA at 37°C for 30 min. DNA electrophoresis was carried out in 2% agarose gel. The gel was stained with ethidium bromide. DNA fragments were visualized by exposing the gel to UV light.

### 2.6. Reverse Transcription-Polymerase Chain Reaction (RT-PCR)

Total RNA was isolated from MA-10 cells using Trizol reagent as recommended by the manufacturer (Invitrogen, Carlsbad, Calif, USA). Reverse transcription was performed in a mixture containing 5 *μ*M random primer, 200 *μ*M dNTP, 2 U/*μ*L MMLV reverse transcriptase together with 5 *μ*L tRNA as the template. The corresponding buffer was performed at 42°C for 90 min followed by 95°C for 10 min. PCR was performed in a mixture containing 2 *μ*L 10X PCR buffer, 0.4 *μ*L 10 mM dNTP, 0.4 *μ*L 20 mM specific forward and reverse primers (primer sequence and corresponding sequence of specific genes were listed in Table 1), 14.7 *μ*L ddH_2_O, 0.1 *μ*L 0.5 U Taq with 2 *μ*L RT product as template for each reaction. Thermocontrolling program was set up as the following: denature at 95°C for 30 sec, annealing at 55°C for 30 sec, elongation at 72°C for 30 sec with another 5 min of elongation at 72°C. The whole mixture was subjected to 30 cycles for amplification of *L19*, 32 cycles for amplification of A_1_-AR, 34 cycles for amplification of A_2A_-AR and A_2B_-AR and 38 cycles for amplification of A_3_-AR. The PCR product was then separated on a 1.5% agarose gel at 120 V for 30 min in 1X TBE buffer (0.09 M Tris, 0.09 M boric acid, 0.001 M EDTA, pH 8.0). The gel was then stained with ethidium bromide for 10 min and destained with mili-Q water. The gel image was captured by using Labwork imager system (Digital CCD Camera, Hamamtsu Photonics system, Bridgewater, USA). 

### 2.7. Radioimmunoassay (RIA)

Media from cultures with different treatments were harvested. Twenty *μ*L of sample was loaded into a glass tube and 100 *μ*L each of progesterone antiserum and ^3^H progesterone were loaded. Equilibrium reaction occurred at 37°C for 30 min and was stopped by putting the tubes in ice. Charcoal was added into the tubes at 4°C for 15 min and centrifuged for 10 min in order to spin down the charcoal-^3^H progesterone complex. The supernatant was poured into 2 mL of scintillation fluid and samples would be counted in a *β*-counter (LS 5000TA, Beckman Inc., Fullerton, Calif, USA).

### 2.8. Immunoblot Analysis

6.0 × 10^5^ cells were cultured in 6 cm dish. After treatment, cells were rinsed with cold PBS. Then, the cells were harvested by 100 *μ*L lysis buffer (20 mM Tris-base, 150 mM NaCl, 1 mM EDTA, 1 mM EGTA, 1% Triton X-100, 2.5 mM Sodium pyrophosphate, 1 mM beta-Glycerolphosphate, 1 mM Na_3_VO_4_). The cell lysate was subjected to centrifugation at 12,000 ×g for 12 min at 4°C. The supernatant was stored at −20°C until used. The protein concentration was determined by Lowry method [24]. Total proteins were separated in 12.5% SDS-PAGE. Electrophoresis was performed in SDS-PAGE running buffer (24 mM Tris/HCl, 0.19 M glycine, 0.5% SDS, pH 8.3). The proteins were transferred to polyvinylidene difluoride membranes (PVDF) in transfer buffer (20 mM Tris/HCl, 150 Mm glycine, 10% methanol, 0.05% SDS). The PVDF membrane with protein on it was incubated in blocking buffer (TBS buffer containing 5% Carnation non-fat dry milk and 0.1% Tween-20) at room temperature for an hour. After washing, the membrane would be incubated in primary antibodies StAR at 1 : 2500 dilutions overnight at 4°C. The membrane would be washed three times (10 min each) with TBS containing 0.1% Tween-20. It was then incubated for an hour at room temperature with fresh blocking buffer containing the secondary antibody, antirabbit IgG. The membrane was washed and the signal detected by using the Renaissance chemiluminescence reagent. The desired protein was quantitated by UVP EC3 Imaging System (Upland, Calif, USA). The amount of *β*-actin in each lane was detected and quantified as well in order to normalize the expression of target protein.

### 2.9. Transient Transfection and Luciferase Assays

The 5′-flanking regions (−1278/+32) of the mouse *StAR *gene were cloned into the *pGL3* basic vector (Promega Corp., Madison, Wis, USA), upstream of a luciferase reporter gene utilizing the *XhoI* and *HindIII* sites. The plasmid *pSV*-Galactosidase control vector (Promega Corp.) was used to normalize transfection efficiency. Plasmids were transfected using Lipofectamine 2000 (Invitrogen 11668-019, Invitrogen Corp., Calif, USA). In brief, 1.2 × 10^5^ cells/500 *μ*L medium were cultured in either 24-well plates to 65–75% confluency and transfected using 0.5 *μ*g of plasmid in the presence of 0.5 *μ*g of *pSV*-Galactosidase control vector. After 6 hr of transfection, MA-10 cells were followed by rising and incubation in serum-free Waymouth medium for 18 hr. After medium was changed, cells were then treated with cordycepin for another 12 hr. Luciferase assays were performed using the Luciferase Reporter Assay System according to the manufacturer's instructions (Promega Corp., Madison, Wis, USA). Briefly, after treatment cells were washed with 1X PBS and 200 *μ*L of the 1X passive lysis buffer (Dual-Luciferase Reporter Assay System, Promega E1910, Promega Corp., Madison, Wis, USA) was added to the plates. The cellular debris was pelleted by centrifugation at 12,000 ×g at 4°C, and the 20 *μ*L supernatant was measured for 50 *μ*L Luciferase Assay Buffer (Dual-Luciferase Reporter Assay System, Promega E1910, Promega Corp., Madison, Wis, USA) in a MiniLumat Luminometer (Turner Designs Corp., Sunnyvale, Calif, USA).

### 2.10. Statistical Analysis

Each data point in the figures represents the mean ± SEM of three or four separate experiments. Statistically significant differences between treatments and controls were determined by one-way ANOVA and then least significance difference (LSD) or with *t*-test comparison procedure. Statistical significance was set at *P* < .05.

## 3. Results

### 3.1. Effects of Cordycepin on Steroidogenesis in MA-10 Mouse Leydig Tumor Cells

**Figure 1:**
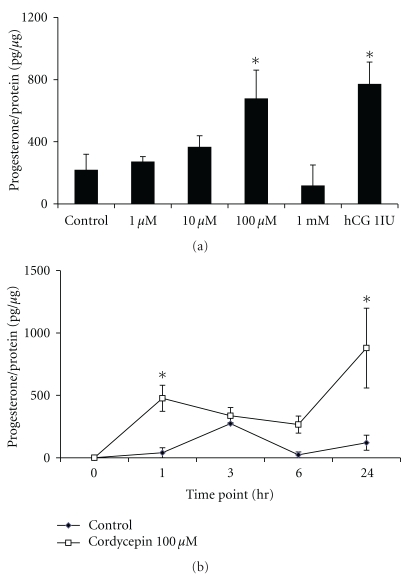
Dose- and time-course effects of cordycepin on steroidogenesis in MA-10 mouse Leydig tumor cells. MA-10 cells (5000 cells/well) were treated with 1, 10, 100 *μ*M and 1 mM cordycepin for 24 hr (a); and MA-10 cells were treated with 100 *μ*M cordycepin for 0, 1, 3, 6, and 24 hr (b), respectively. Treatment of hCG (1 IU) was used as a positive control. Media were collected and assayed for progesterone production by RIA. Each data point in the figure represents the mean the mean ± SEM of three independent experiments with triplicates in each treatment. Asterisk *(*P* < .05) above the bars indicates significant difference compared with control.

To test the hypothesis that cordycepin influences the production of steroid hormone in MA-10 mouse Leydig tumor cells; we initially determined the effect of cordycepin on the production of progesterone. MA-10 cells were incubated with different dosages (1, 10, 100 *μ*M, and 1 mM) of cordycepin for 24 hr. Results showed that the progesterone production induced by 100 *μ*M cordycepin was more than 3 folds significantly compared to the control (217.5 ± 101.7 versus 675.9 ± 185.4 pg/*μ*g protein; *P* < .05) (Figure 1(a)). As shown in Figure 1(b), cordycepin at 100 *μ*M significantly stimulated progesterone to a maximum at 24 hr (*P* < .05). According to the results, 100 *μ*M cordycepin for 24 hr treatment was used to investigate the possible cellular mechanism.

### 3.2. RT-PCR Analysis for Detection of A_1_, A_2A_, A_2B_, and A_3_ Adenosine Receptor mRNA Transcripts in MA-10 Mouse Leydig Tumor Cells

**Figure 2:**
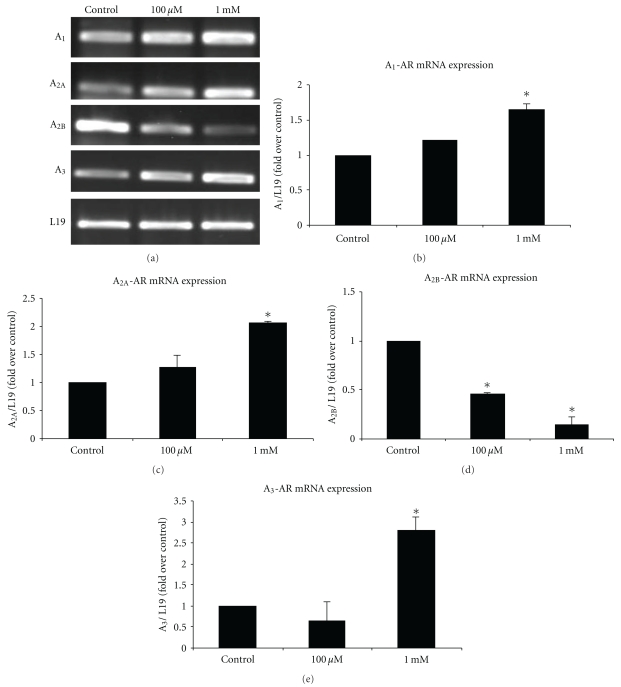
Characterization of A_1_, A_2A_, A_2B_, and A_3_ AR mRNA expression in MA-10 mouse Leydig tumor cells. MA-10 cells (1× 10^6^) were cultured in Waymouth medium to 80% confluence, and then the total RNA were extracted and the expression of adenosine receptor subtypes mRNA were analyzed by RT-PCR. (a) shows A_1_, A_2A_, A_2B_, and A_3_ AR mRNA expression in response to cordycepin at different dosages (100 *μ*M and 1 mM) for 24 hr. *L19* is the housekeeping gene used as an internal control. (b) shows the integrated optical density (IOD) of A_1_, A_2A_, A_2B_, and A_3_ AR mRNA expression after normalization with *L19*. Each data point in the figure represents the mean ± SEM of three separate experiments. Asterisk *(*P* < .05) above the bars indicates significant difference compared with control.

It has been reported that cordycepin could inhibit lung carcinoma and melanoma cell growth by stimulating A_3_-AR [25, 26]. We hypothesized for the presence of functional adenosine receptor subtypes that involved in cordycepin-induced steroidogenesis in MA-10 cells. Thus, mRNA expressions of all four AR subtypes, A_1_, A_2A_, A_2B_, and A_3_, with cordycepin treatment in MA-10 cells were analyzed by RT-PCR. The results showed that cordycepin (100 *μ*M and 1 mM) could change the mRNA expression among four AR subtypes (Figure 2(a)). The normalized results showed that the treatment of 1 mM cordycepin for 24 hr upregulated the expression of A1-AR mRNA for about 1.5 fold (*P* < .05) (Figure 2(b)). Cordycepin (1 mM) upregulated the expression of A_2A_-AR mRNA for about 2 folds (*P* < .05) (Figure 2(c)). Cordycepin at 1 mM would also upregulate the expression of A_3_-AR mRNA for about 3 folds (*P* < .05) (Figure 2(e)). However, both 100 *μ*M and 1 mM cordycepin would downregulate A_2B_-AR mRNA expression by 60% and 80%, respectively (*P* < .05) (Figure 2(d)).

### 3.3. A_1_, A_2A_, A_2B_, and A_3_ AR Were Involved in Cordycepin-Induced Steroidogenesis in MA-10 Mouse Leydig Tumor Cells

**Figure 3:**
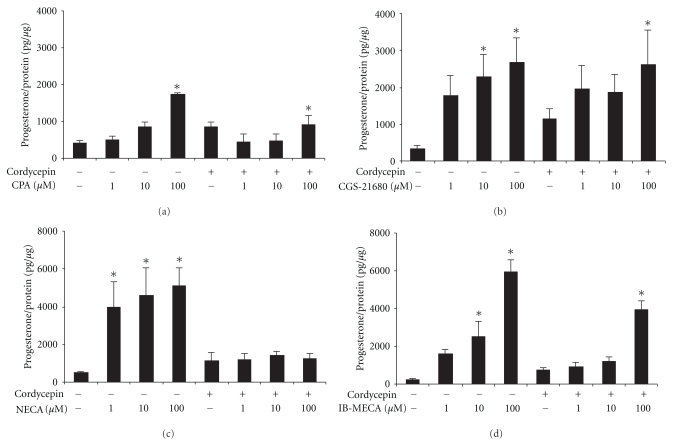
A_1_, A_2A_, A_2B_, and A_3_ AR are involved in cordycepin-induced steroidogenesis. MA-10 cells (5000 cells/well) were treated with different dosage (1~100 *μ*M) of A_1_, A_2A_, A_2B_, and A_3_ AR agonists, which were (a) CPA, (b) CGS-21680, (c) NECA, and (d) IB-MECA in the absence or presence of 100 *μ*M cordycepin for 24 hr. Media were collected and assayed for progesterone production by RIA. Each data point in the figure represents the mean the mean ± SEM of three independent experiments with triplicates in each treatment. Asterisk *(*P* < .05) above the bars indicates significant difference compared with control.

**Figure 4:**
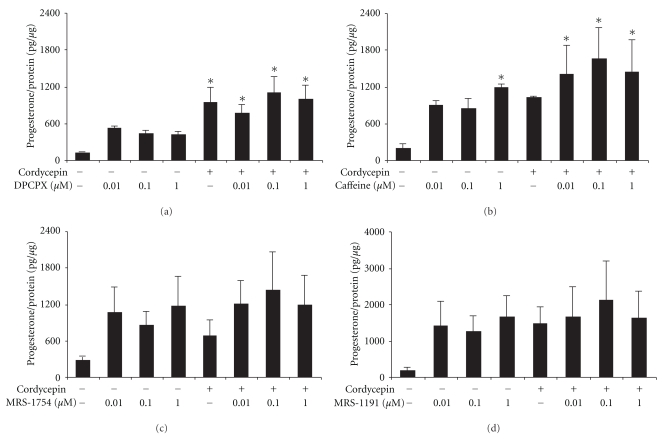
Effects of AR antagonists on cordycepin-induced steroidogenesis in MA-10 mouse Leydig tumor cells. MA-10 cells (5,000 cells/well) were treated with different dosage (10 nM~1 *μ*M) of A_1_, A_2A_, A_2B_, and A_3_ AR antagonists, which were (a) DPCPX, (b) Caffeine, (c) MRS-1754, and (d) MRS-1191 in the absence or presence of 100 *μ*M cordycepin for 24 hr. Media were collected and assayed for progesterone production by RIA. Each data point in the figure represents the mean the mean ± SEM of three independent experiments with triplicates in each treatment. Asterisk *(*P* < .05) above the bars indicates significant difference compared with control.

By using AR agonists (1~100 *μ*M) to treat MA-10 cells for 24 hr, the results showed that A_1_-, A_2A_-, A_2B_-, and A_3_-AR agonists could stimulate progesterone production in MA-10 mouse Leydig cells (*P* < .05) (Figures 3(a)–3(d)). Compared to the control, A_1_-AR agonist could significantly stimulate progesterone production and reach 4.1 folds by 100 *μ*M CPA (*P* < .05) (Figure 3(a)). A_2A_-AR agonist could significantly stimulate progesterone production and reach 6.8 and 7.9 folds by 10 and 25 *μ*M CGS-21680, respectively, (*P* < .05) (Figure 3(b)). A_2B_-AR agonist could significantly stimulate progesterone production and reach 7.6, 8.9, and 9.8 folds by 1, 10, and 100 *μ*M NECA, respectively, (*P* < .05) (Figure 3(c)). A_3_-AR agonist could significantly stimulate progesterone production and reach 10.5 and 24.7 folds by 10 and 50 *μ*M IB-ECA, respectively (*P* < .05) (Figure 3(d)). On the other hand, cordycepin (100 *μ*M) cotreated with A_1_-AR agonist would significantly decrease CPA-stimulated (100 *μ*M) progesterone production by 47% (*P* < .05) (Figure 3(a)). Cordycepin (100 *μ*M) cotreated with A_2B_-AR agonist would significantly decrease NECA-stimulated (1, 10 and 100 *μ*M) progesterone production by 70%, 69%, and 47%, respectively, (*P* < .05) (Figure 3(c)). Cordycepin (100 *μ*M) cotreated with A_3_-AR agonist would significantly decrease IB-MECA (50 *μ*M)-stimulated progesterone production by 33% (*P* < .05) (Figure 3(d)). However, cordycepin (100 *μ*M) cotreated with A_2A_-AR agonist would not affect CGS-21680-stimulated progesterone production (*P* > .05) (Figure 3(b)). These results demonstrated that cordycepin could regulate AR subtype expressions to stimulate progesterone production in MA-10 cells. To further understand the mechanism of functional AR subtypes that involved in the cordycepin-induced steroidogenesis in MA-10 mouse Leydig tumor cells, selective A_1_-, A_2A_-, A_2B_-, and A_3_-AR subtype antagonists (10 nM~1 *μ*M) were used to cotreat with cordycepin for 24 hr. The results showed that A_1_-, A_2A_-, A_2B_-, and A_3_-AR antagonists did not have inhibitory effect on cordycepin-induced progesterone production in MA-10 cells (*P* > .05) (Figures 4(a)–4(d)).

### 3.4. Effects of Cordycepin on StAR Protein and Promoter Expressions in MA-10 Mouse Leydig Tumor Cells

**Figure 5:**
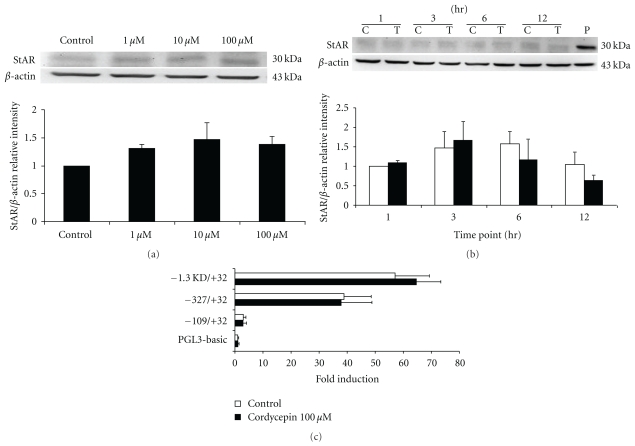
Effects of cordycepin on the expression of StAR protein and *StAR *promoter activity in MA-10 mouse Leydig tumor cells. MA-10 cells were treated with 1, 10, and 100 *μ*M cordycepin for 3 hr (a); and MA-10 cells were treated with 100 *μ*M cordycepin for 1, 3, 6, and 12 hr (b), respectively. Treatment of hCG (1 IU) was used as a positive control. The expression of StAR (30 kDa) and *β*-actin (43 kDa) detected by Western blotting. Quantitative analyses of the levels of StAR and *β*-actin (a, b) (*n* = 3) are shown. Asterisk *(*P* < .05) above the bars indicates significant difference compared with control. Serial deletion constructs of the *StAR *promoter were transiently transfected into MA-10 cells (c). The promoter activity was calculated by dividing firefly signals to Renilla signals. Each data point in the figure represents the mean ± SEM of three independent experiments.

It has been previously shown that steroidogenesis induced by* Cordycep sinensis* in MA-10 cells requires de novo protein synthesis [27]. To further understand the mechanism of cordycepin-induced steroidogenesis in MA-10 cells, StAR protein expression was investigated by Western blotting analysis. MA-10 cells were incubated with different dosages (1~100 *μ*M) of cordycepin for 3 hr or 100 *μ*M cordycepin for 1, 3, 6, and 12 hr, respectively. Results showed that the expression of StAR protein was not activated by cordycepin with different dosages (Figure 5(a)) and different time treatments (Figure 5(b)) in MA-10 mouse Leydig tumor cells (*P* > .05). 

In addition, MA-10 cells were transiently transfected with plasmid containing luciferase gene with 5′-flanking *StAR* promoter constructs between −1.3 k to +32 regions, and cordycepin (100 *μ*M) was added for 12 hr treatment. Results of 5′-deletion promoter analysis indicated that cordycepin did not increase *StAR* promoter activity (*P* > .05) (Figure 5(c)). Taken together, these results suggest that StAR protein and *StAR *promoter might not be involved in the cordycepin-induced steroidogenesis in MA-10 cells.

### 3.5. Effects of Cordycepin and Adenosine on Cell Death in MA-10 Mouse Leydig Tumor Cells

**Figure 6:**
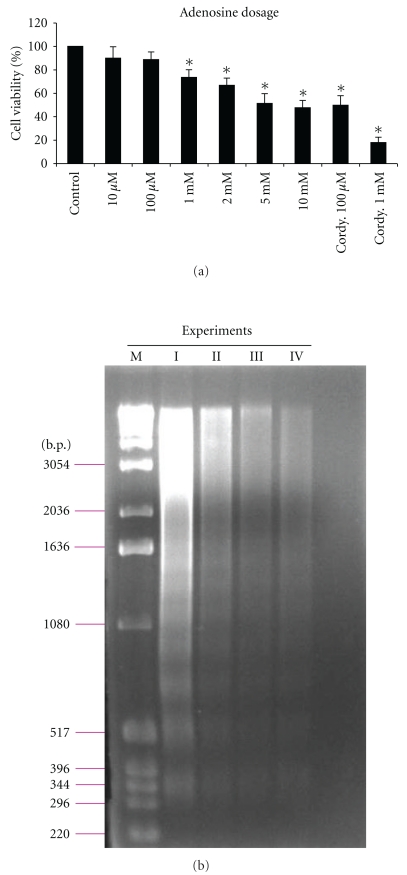
Effects of cordycepin and adenosine on cell death in MA-10 mouse Leydig tumor cells. MA-10 cells were cultured in medium without or with adenosine (10 *μ*M~10 mM), 100 *μ*M and 1 mM cordycepin for 48 hr (a). MTT assay was performed to assess cell viability. Data represent the mean ± SEM of four separate experiments. Asterisk *(*P* < .05) above the bars indicates significant difference compared with control. (cordy. = cordycepin). (b) Gel electrophoresis of a 1 kb DNA ladder marker (lane M) or DNA isolated from MA-10 cells that were cultured in the presence of 10 mM adenosine for 48 hr. DNA was visualized by ethidium bromide staining and photographed under UV illumination. Experiments were performed four times (Exp. I–IV) with similar results.

According to our previous studies, cordycepin has antitumor activity in OECM-1 oral cancer and MA-10 mouse Leydig tumor cell lines [3, 28]. We hypothesized that cordycepin, an adenosine analogue, might act through AR to induce cell death in MA-10 mouse Leydig tumor cells. Thus, the death effects of cordycepin and adenosine on MA-10 cell line was investigated by culturing MA-10 cells with different dosage of adenosine (10 *μ*M~10 mM) and cordycepin (100 *μ*M~1 mM) for comparison. MTT assay showed that 1, 2, 5, and 10 mM adenosine significantly decreased MA-10 cell viability to 78%, 65%, 43%, and 39% (*P* < .05), and cordycepin (100 *μ*M and 1 mM) significantly decreased MA-10 cell viability to 44% and 13% (*P* < .05) for 48 hr treatment, respectively (Figure 6(a)). DNA fragmentation was also observed in the MA-10 cells with 10 mM adenosine after 48 hr treatment (Figure 6(b)). The results are comparable regarding the cell death effects between cordycepin and adenosine on MA-10 cells.

### 3.6. A_1_, A_2A_, A_2B_, and A_3_ AR Were Involved in Cordycepin-Induced Cell Death in MA-10 Mouse Leydig Tumor Cells

**Figure 7:**
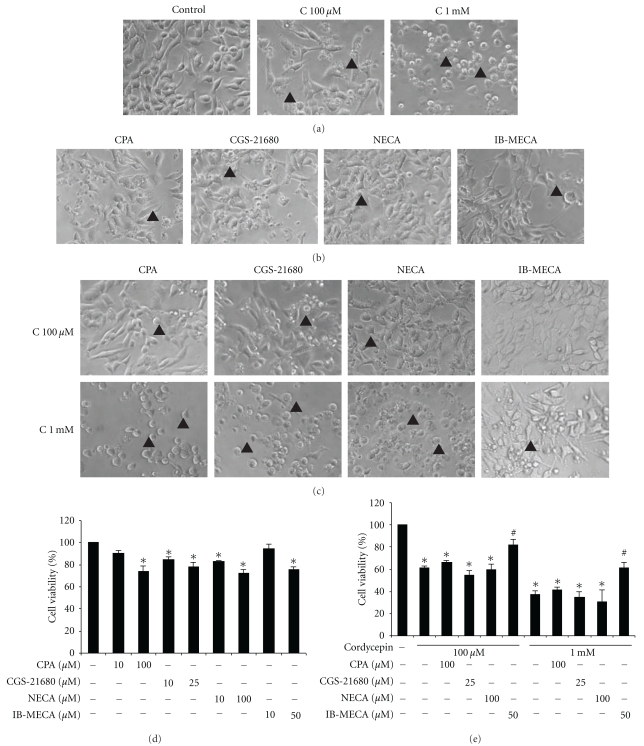
A_1_, A_2A_, A_2B_, and A_3_ AR were involved in cordycepin-induced cell death in MA-10 mouse Leydig tumor cells. MA-10 cells were treated with A_1_, A_2A_, A_2B_, and A_3_ AR agonists in the absence or presence of cordycepin for 24 hr. (a) shows that MA-10 cells were incubated in the absence or presence of cordycepin (100 *μ*M and 1 mM) for 24 hr. (b) shows that MA-10 cells were incubated in AR agonists (A_1_ 100 *μ*M, A_2A_ 25 *μ*M, A_2B_ 100 *μ*M, and A_3_ 50 *μ*M) for 24 hr. (c) shows that MA-10 cells were incubated in cordycepin (100 *μ*M or 1 mM) plus AR agonists (A_1_ 10 *μ*M, A_2A_ 10 *μ*M, A_2B_ 10 *μ*M, and A_3_ 50 *μ*M) for 24 hr. Cell morphology was observed and recorded under light microscopy (Olympus, CK 40). Note the same magnification (200x) among Figures. Arrowhead (▲) indicates rounded-up cells and/or membrane-blebbed cells. MA-10 cells (5,000 cells/well) were treated with different dosages (1~100 *μ*M) of A_1_-, A_2A_-, A_2B_-, and A_3_-AR agonist in the absence (d) or presence of cordycepin (100 *μ*M and 1 mM) for 24 hr (e). MTT assay was used to assess cell viability, which was proportional to absorbance at 590 nm. Each data point in the Figure represents the mean the mean ± SEM of four independent experiments with triplicates in each treatment. Asterisk *(*P* < .05) above the bars indicates significant difference compared with control. ^#^(*P* < .05) above the bars indicates significant difference compared with cordycepin alone treatment group (C: cordycepin).

Previous data demonstrate that expressions of AR subtypes, A_1_, A_2A_, A_2B_, and A_3_ mRNA are found in MA-10 cells, and their expression could be modulated by the presence of cordycepin. To investigate whether cordycepin-induced apoptosis was mediated via adenosine receptors on MA-10 mouse Leydig tumor cells, cells were treated with A_1_-, A_2A_-, A_2B_-, and A_3_-AR agonists (CPA, CGS-21680, NECA and IB-MECA) for 24 hr, respectively. Results show that some MA-10 cells appeared rounded-up phenomenon with floating and membrane-blebbing after 24 hr cordycepin (100 *μ*M and 1 mM) treatment (Figure 7(a)). In addition, MA-10 cells treated with A_1_-, A_2A_-, A_2B_-, and A_3_-AR agonists (100 *μ*M, 25 *μ*M, 100 *μ*M, and 50 *μ*M, resp.) for 24 hr also induced cells to have rounded-up phenomenon with floating cells (Figure 7(b)). Cotreatment of cordycepin (100 *μ*M and 1 mM) with A_1_-, A_2A_-, and A_2B_-agonists (100 *μ*M, 25 *μ*M, and 100 *μ*M, resp.) for 24 hr induced cells to have rounded-up phenomenon with floating and membrane-blebbing. Interestingly, A_3_-AR agonist (50 *μ*M) cotreated with cordycepin (100 *μ*M and 1 mM) for 24 hr would let some MA-10 cell recover back to polygonal shape, which was similar to the control group (Figure 7(c)). MTT cytotoxicity assay was further employed to assess cell viability (Figure 7(d)). Compared to the control, A_1_-AR agonist, CPA, at 100 *μ*M caused a significant reduction in cell viability to 73.8 ± 5.2% (*P* < .05). A_2A_-AR agonist, CGS-21680, at 10 *μ*M and 25 *μ*M caused a significant reduction in cell viability to 84.9 ± 2.4% and 78.4 ± 4.0%, respectively (*P* < .05). A_2B_-AR agonist, NECA, at 10 *μ*M and 100 *μ*M caused significant reductions in cell viability to 82.9 ± 1.5% and 72.9 ± 2.6%, respectively (*P* < .05). A_3_-AR agonist, IB-MECA, at 50 *μ*M caused a significant reduction in cell viability to 75.7 ± 2.4% (*P* < .05). These results showed that A1-, A_2A_-, A_2B_- , and A3-AR agonists significantly reduced MA-10 cell viability, respectively (Figure 7(d)) (*P* < .05). Besides, cordycepin alone (100 *μ*M and 1 mM) significantly decreased MA-10 cell viability approximately to 60% and 40%, respectively, after 24 hr treatment (Figure 7(e)) (*P* < .05). However, A_1_-, A_2A_- , and A_2B_-AR agonists plus cordycepin treatments did not have any additive effects on MA-10 cell death, respectively (Figure 7(e)) (*P* > .05). But, A_3_-AR agonist (50 *μ*M) cotreated with cordycepin (100 *μ*M or 1 mM) would reverse MA-10 cell viability back to 81.6 ± 9.6% or 61.6 ± 7.8%, respectively (Figure 7(e)). These results showed that A_1_- , A_2A_-, A_2B_-, and A_3_-AR might be very important regarding the antitumor effects of cordycepin in MA-10 cells. 

### 3.7. AR Antagonists Could Prevent Cordycepin-Induced Cell Death in MA-10 Mouse Leydig Tumor Cells 

**Figure 8:**
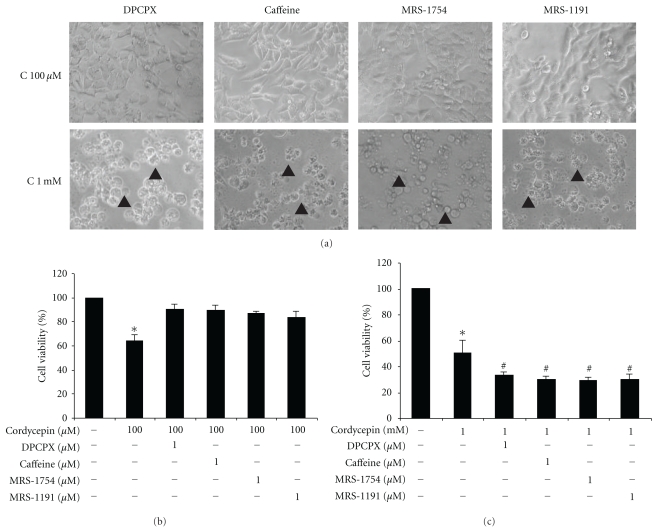
Effects of AR antagonists on cordycepin-induced cell death in MA-10 mouse Leydig tumor cells. MA-10 cells were treated with different adenosine receptor antagonists in the absence or presence of cordycepin for 24 hr. (a) shows that MA-10 cells were incubated in cordycepin (100 *μ*M or 1 mM) plus 1 *μ*M selective AR antagonists for 24 hr. Cell morphology was observed and recorded under light microscopy (Olympus, CK 40). Note the same magnificantion (200×) among Figures. Arrowhead (▲) indicate rounded-up cells and/or membrane-blebbed cells. MA-10 cells (5,000 cells/well) were treated with different dosages (1~100 *μ*M) of A_1_-, A_2A_-, A_2B_-, and A_3_-AR antagonists in the presence of 100 *μ*M (b) or 1 mM (c) cordycepin for 24 hr. MTT assay was used to assess cell viability, which was proportional to absorbance at 590 nm. Each data point in the Figure represents the mean the mean ± SEM of four independent experiments with triplicates in each treatment. Asterisk *(*P* < .05) above the bars indicates significant difference compared with control. ^#^(*P* < .05) above the bars indicates significant difference compared with cordycepin alone treatment group (C: cordycepin).

In order to confirm whether cordycepin-induced apoptosis was mediated via AR on MA-10 mouse Leydig tumor cells,  cells were treated with cordycepin  (100 *μ*M and 1 mM) plus A_1_- ,   A_2A_-,  A_2B_-, and A_3_-AR antagonists (DPCPX, Caffeine, MRS1754 and MRS1191) for 24 hr, respectively.  All A_1_-, A_2A_-,  A_2B_ , and A_3_-AR antagonists could let cordycepin-treated MA-10 cells recover back to polygonal shape, which is similar to the cells in control group (Figure 8(a)). In MTT assay, the cotreatment of 100 *μ*M  cordycepin  with 1 *μ*M  A_1_- , A_2A_-, A_2B_-, and A_3_-AR antagonists could also significantly reverse cell viability to 90.6%  ± 3.8%, 89.2%  ± 4.8%, 86.7%  ± 1.9% and 83.3%  ± 5.7%., respectively (*P* < .05) (Figure 8(b)). However, the cotreatment of 1 mM cordycepin with A_1_-, A_2A_-, A_2B_-, and A_3_-AR antagonists did not reverse cell viability (Figure 8(c)). These results indicate that the apoptotic effects of cordycepin on MA-10 cells could be mediated by adenosine receptors.

## 4. Discussion

In this study, we demonstrate that cordycepin, a pure substance isolated from the *Cordyceps sinensis*, could stimulate steroidogenesis in MA-10 mouse Leydig tumor cells. Besides, cordycepin could induce the antitumor effect possibly through adenosine receptors in MA-10 mouse Leydig tumor cells.

First, we found that cordycepin increased the expression of A_1_-, A_2A_-, and A_3_-AR mRNA but decreased the expression of A_2B_-AR mRNA at 24 hr treatment. Under microscopic observation, we found that cordycepin-treated MA-10 cells exhibited cellular shrinkage and membrane blebbing, and finally cells detached from the dish. By MTT and DNA ladder assays, adenosine also significantly reduced MA-10 cell viability in a dose-dependent manner and induced DNA fragmentation. The effective concentration (EC50) of adenosine which could cause 50% inhibition of MA-10 cells growth was 5 mM after 48 hr treatment. However, we observed that the death effect of cordycepin was somewhat greater than adenosine in the same concentration (100 *μ*M and 1 mM). These phenomena indicate that the apoptotic effect by adenosine in MA-10 cells was comparable.

Nakamura and coworkers have demonstrated that cordycepin inhibited lung carcinoma cells and melanoma cells growth by stimulating A_3_-AR [25, 26]. Many evidences have also illustrated that A_1_- and A_2A_-AR expression mediated cell death by inducing apoptosis in breast carcinoma cells, astrocytoma cells, mouse thymocytes [29–31]. In this study, we showed that cordycepin could significantly stimulate the expression of A_3_-AR mRNA in cordycepin-treated MA-10 cells. Moreover, this effect also occurred in A_1_-AR and A_2A_-AR mRNA expressions. In addition, we also demonstrated that cordycepin induced MA-10 mouse Leydig tumor cell apoptosis through caspase-9 and caspase-3 pathways [28]. These data suggested that AR might participate in cordycepin-induced apoptosis pathway in MA-10 mouse Leydig cells. 

We continued to investigate the AR agonists to cotreat with cordycepin in MA-10 cells. We found that A_3_-AR agonists could significantly rescue cordycepin-induced apoptosis in MA-10 cells. Many reports indicate that adenosine displays contradictory effects such as the induction of cell apoptosis or stimulation of cell proliferation [32, 33]. It has been reported that the A_3_-AR agonist (CL-IB-MECA) reduced apoptosis in human astroglioma D384 cells [34]. In fact, some evidences have shown that adenosine via A_1_-AR- and A_3_-AR-mediated cytoprotection involves phospholipase C, PKC, and p38 MAPK pathways, and reduced ROS production in cardiomyocytes [35–37]. In this study, we demonstrated that activation of AR would induce apoptosis in MA-10 cells. However, activation of different subtypes of AR could trigger apoptosis/survival pathway in MA-10 cells, which must be further investigated.

Recent reports demonstrated that adenosine receptor antagonists can resume cell viability on toxicant drug-induced apoptosis in thyroid cancer cells [38, 39]. We used the selective AR antagonists to cotreat with cordycepin in  MA-10  cells. We also demonstrated that A_1_-, A_2A_-, A_2B_-  , and A_3_-AR antagonist could significantly rescue 100 *μ*M cordycepin-induced apoptosis in MA-10 cells. These results indicate that the apoptotic effects of cordycepin on MA-10 cells could be mediated by AR. However, 1 mM cordycepin cotreated with adenosine antagonists could not reverse cell viability, but even promote cell death. Recent developments of potent and selective antagonists of AR subtypes have been valuable for further defining the physiological effects of the various AR subtypes. Moreover, there are substantial species differences in the affinity of these compounds, and these selective compounds may fairly potent as antagonists of another AR subtypes [40].

It has been shown that adenosine-stimulated steroidogenesis might be involved in the A_2A_- and A_2B_-AR and phosphorylation of MAPK ERK 1/2 signal pathway in rat adrenal cells [41]. Moreover, it is proposed that ligand binding results in a change in receptor state from an inactive to an active state will ultimately elicit its biological response based on the receptor's conformation [42]. In this model, agonists are thought to have selective binding affinity for the preexisting resting and active states or can induce a conformational change to a different receptor state and effects binding affinity of a ligand [43]. In the present study, cordycepin cotreated with A_2B_-AR agonist (NECA) would significantly decrease NECA-stimulated progesterone production. It is probable that cordycepin may bind with other receptors and induce conformation change to effect binding affinity of A_2B_-AR agonist. This result is consistent with the observation that cordycepin (100 *μ*M) decreased the expression of A_2B_-AR mRNA at 24 hr treatment. Although cordycepin (100 *μ*M) did not affect the expression of A_1_- and A_3_-AR mRNA, cordycepin cotreated with A_1_- and A_3_-AR agonist (CPA and IB-MECA) also significantly decreased their progesterone production. On the other hand, cordycepin did not affect the A_2A_-AR agonist (CGS-21680)-stimulated progesterone production. It is reasonable that cordycepin may compete with CGS-21680 in the same biding site of A_2A_-AR to stimulate progesterone production. Here, we demonstrated that activation of A_1_-, A_2A_-, A_2B_-, and A_3_-AR could induce steroidogenesis in MA-10. However, we used the AR antagonists to cotreat with cordycepin, and results illustrated huge variations. AR antagonists only slightly increased progesterone production than control group in MA-10 cells, which may decrease the accuracy. More experiments should be conducted to decrease the inconsistence.

Steroid production in Leydig and granulosa cells are regulated by various factors, including cAMP/PKA-dependent [44], mitogen-activated protein kinase/extracellular signal-regulated kinase 1/2 (MAPK ERK 1/2) [45, 46], and protein kinase C (PKC) signaling pathways [47, 48]. These kinases modulate cellular processes by phosphorylations. The family of MAPKs includes extracellular regulating kinase (ERK), c-Jun NH(2)-terminal kinase (JNK), and p38, with each MAPK signaling pathway consisting of at least three components, a MAPK kinase kinase (MAP3k), a MAPK kinase (MAP2k), and a MAPK. Some reports have shown the stimulatory effects of MAPK ERK 1/2 on steroidogenesis [49, 50], and the others have demonstrated the inhibitory effects on steroidogenesis [51, 52]. PKC signaling pathway can be activated by signals such as increases in the concentration of diacylglyceride (DAG), phospholipase C (PLC), or Ca^2+^ [53]. Recent reports indicate that *DAX-1 *gene interplay between the PKA/PKC signaling pathways and represses expression of StAR protein and steroidogenesis [54]. 

It is well established that StAR protein is essential for steroidogenesis, and that ERK 1/2 phosphorylation driven by mitochondrial PKA will induce StAR protein expression and then steroidogenesis [46]. The previous data showed that cordycepin upregulates the expression of StAR mRNA and StAR protein to induce steroidogenesis through the PKA signaling pathway in normal mouse Leydig cells [55]. It has also been documented that the downstream effectors of PKA signaling pathway include several transcription factors [56]. However, our results indicated that cordycepin could not induce StAR protein and *StAR *promoter expressions in MA-10 cells. Our data are somewhat inconsistent to several studies that steroidogenesis could be trigger by cAMP and StAR-independent pathways [57–59]. Therefore, it is possible that the cordycepin could possibly acitvate MAPK-ERK 1/2 and PKC pathways without increasing of *StAR *promoter and StAR protein expression to induce steroidogenesis in MA-10 cells, which will be worth to further investigate.

In conclusion, the present studies demonstrate that cordycepin is one of the active constituent of *Cordyceps sinensis*, which can stimulate progesterone production in MA-10 cells. Meanwhile, cordycepin could also activate AR and simultaneously induce steroidogenesis and apoptosis in MA-10 mouse Leydig tumor cells.

## Figures and Tables

**Table 1:** Sequence of primers used for reverse transcription-polymerase chain reaction (RT-PCR).

	Sequence (5′ to 3′)	PCR size
L19	F GAA ATC GCC AAT GCC AAC TC R TCT TAG ACC TGC GAG CCT CA	405 b.p.
A_1_-AR	F CGG GAT CCT ACA TCT CGG CCT TCC AGG R GGA ATT CAG TAG GTC TGT GGC CCA ATG	219 b.p.
A_2A_-AR	F CGG GAT CCG TCC CTG GCC ATC ATC GT R GGA ATT CGA TCC TGT AGG CGT AGA T	177 b.p.
A_2B_-AR	F CGG GTA CCC CTC GAG TGC ATT ACA GA R CCG CCG AAA CCT TTA TAC CTG AGC	216 b.p.
A_3_-AR	F CGG GAT CCC GTT CCG TGG TCA GTT TGR GGA ATT CGC AGG CGT AGA CAA TAG G	384 b.p.
